# HyperArc^TM^ volumetric modulated arc therapy for hypopharyngeal cancer with solitary recurrence in the cervical vertebra: A case report and literature review

**DOI:** 10.1097/MD.0000000000038427

**Published:** 2024-06-07

**Authors:** Chia-Hui Lin, Jenny Que, Sheng-Yow Ho

**Affiliations:** a Department of Radiation Oncology, Chi Mei Medical Center, Tainan, Taiwan; b Center of General Education, Chia Nan University of Pharmacy and Science, Tainan, Taiwan.

**Keywords:** HyperArc^TM^, hypopharyngeal cancer, reirradiation, volumetric modulated arc therapy

## Abstract

**Rationale:**

It is difficult to reirradiate head and neck cancers because of the toxicity from previous radiation dose delivery. Conventional volumetric modulated arc therapy (VMAT) and intensity-modulated radiation therapy often have poor target coverage. The new HyperArc^TM^ VMAT (HA-VMAT) planning approach reportedly has better target coverage, higher conformity, and can spare normal organs compared to conventional VMAT; however, research on recurrent head and neck cancers is limited. Here, we report the clinical outcomes of HA-VMAT for previously irradiated hypopharyngeal cancer with solitary recurrence in the first cervical vertebra (C1).

**Patient concerns:**

A 52-year-old Asian male was diagnosed with a hypopharyngeal cancer. The patient received concurrent chemoradiotherapy with a radiation dose of 70 Gy in 33 fractions and achieved complete clinical response. Two years later, solitary recurrence was observed in the C1 vertebra.

**Diagnoses:**

Solitary recurrence in the C1 vertebra.

**Interventions:**

Owing to concerns regarding the toxicity to adjacent organs, we decided to use HA-VMAT to achieve better tumor coverage and critical organ sparing.

**Outcomes:**

Tumor regression was observed on the imaging. At 9 months follow-up, the patient was disease-free and had no late toxicities.

**Lessons:**

This is the first report regarding the clinical outcomes of HA-VMAT for previously irradiated hypopharyngeal cancer with solitary recurrence over the C1 vertebra. HA-VMAT achieves highly conformal dose distribution and excellent sparing of critical organs. There was a favorable initial clinical response with no toxicity. Long-term follow-up is essential in such cases.

## 1. Introduction

Reirradiation for head and neck cancer is often difficult because of the toxicity from previous radiation dose delivery in critical organs, such as the brainstem or spinal cord. Therefore, it is important to develop techniques that minimize radiation delivery to nearby critical organs. Recently, the new HyperArc^TM^ volumetric modulated arc therapy (HA-VMAT) planning approach has provided higher conformity and more rapid dose fall-off than conventional VMAT (conformity index: 0.93 vs 0.90, *P* = .01; gradient index: 3.06 vs 3.91, *P* < .01).^[1]^ In the conventional VMAT planning, the number of isocenter, beam angle, and couch rotation angle are manually selected. In the HA-VMAT planning, the position of isocenter is determined automatically based on the selected targets. The field size and collimator angle are optimized to reduce irradiation of normal organs. One coplanar full arc and 3 noncoplanar partial arcs are arranged automatically. The optimization algorithm is stereotactic radiosurgery (SRS) normal tissue objective, which generates plans that have steep dose decay in space from high-dose level to low-dose level. The HA-VMAT planning has better optimization process than conventional VMAT. Therefore, HA-VMAT results in superior target conformity and better normal tissue sparing. HA-VMAT has previously been used for radiosurgery of brain metastases.^[2]^ In the treatment of scalp angiosarcoma, HA-VMAT provided significantly lower mean brain doses than conventional VMAT for the treatment of scalp angiosarcoma (12.63 ± 3.31 Gy vs 17.11 ± 5.25 Gy, *P* = .005).^[3]^ Another study revealed that HA-VMAT plans achieved better tumor coverage in maxillary sinus carcinoma than did conventional VMAT plans (D_99%_: 62.7 ± 2.1 Gy vs 61.9 ± 2.4 Gy, *P* = .009).^[4]^ Most of these studies, however, only reported the superior planning of HA-VMAT; clinical outcomes among patients with head and neck cancer treated with HA-VMAT were scarce. Here, we report the clinical outcomes of HA-VMAT for previously irradiated hypopharyngeal cancer with solitary recurrence in the first cervical vertebra (C1).

## 2. Case report

### 2.1. Clinical course

**Figure 1.**
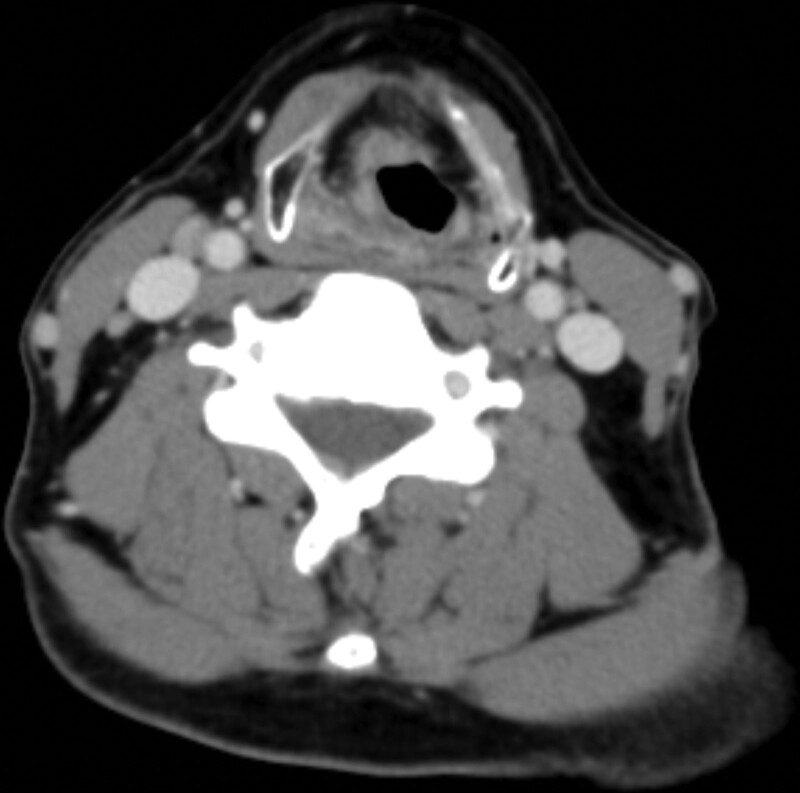
Tumor was seen over the right hypopharynx on CT scan. CT = computed tomography.

**Figure 2.**
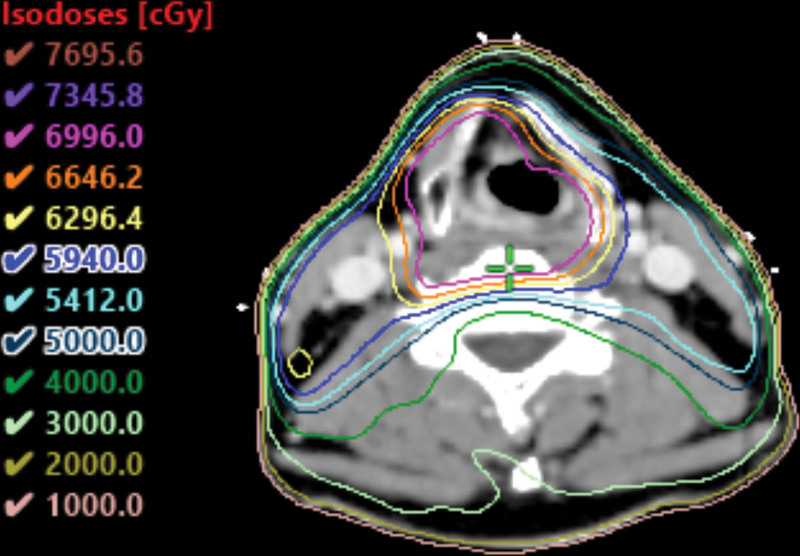
The planning of first course of radiotherapy.

**Figure 3.**
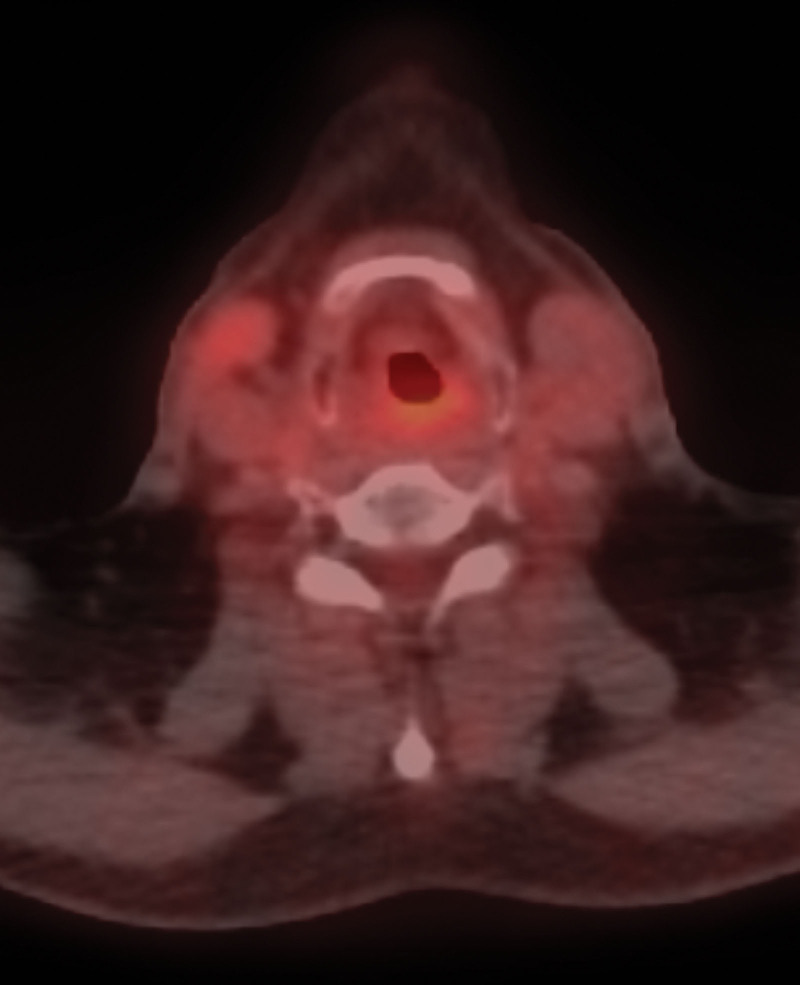
PET CT scan revealed no evidence of FDG-avid malignant lesions. CT = computed tomography, FDG = fluorodeoxyglucose, PET = positron emission tomography.

**Figure 4.**
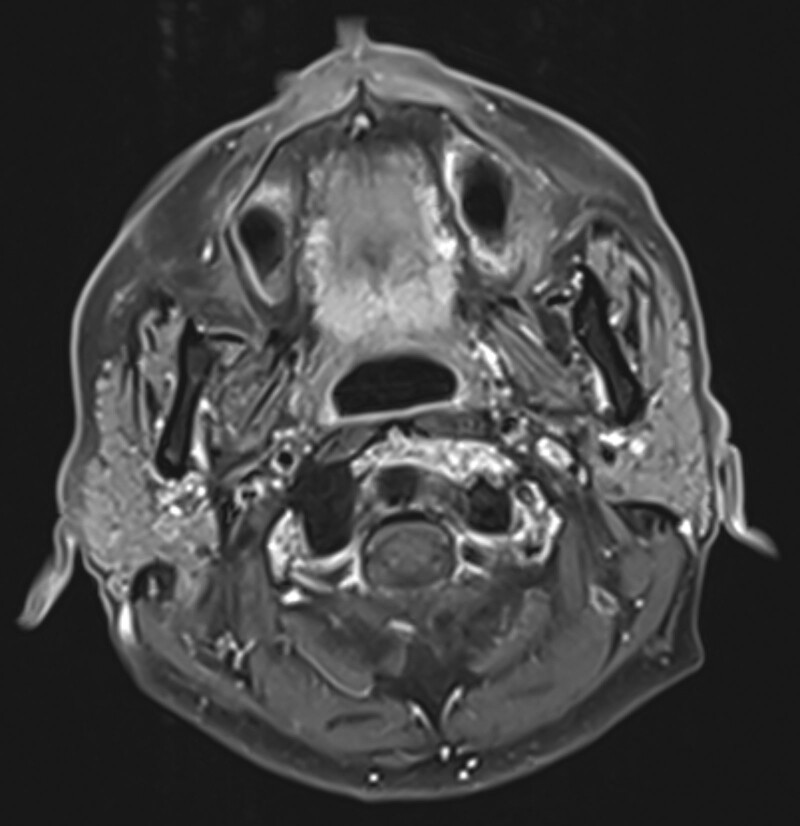
MRI showed a solitary enhancing lesion with normal marrow replacement and cortical bone breakthrough in the left anterior arch of the C1 vertebra. MRI = magnetic resonance imaging.

**Figure 5.**
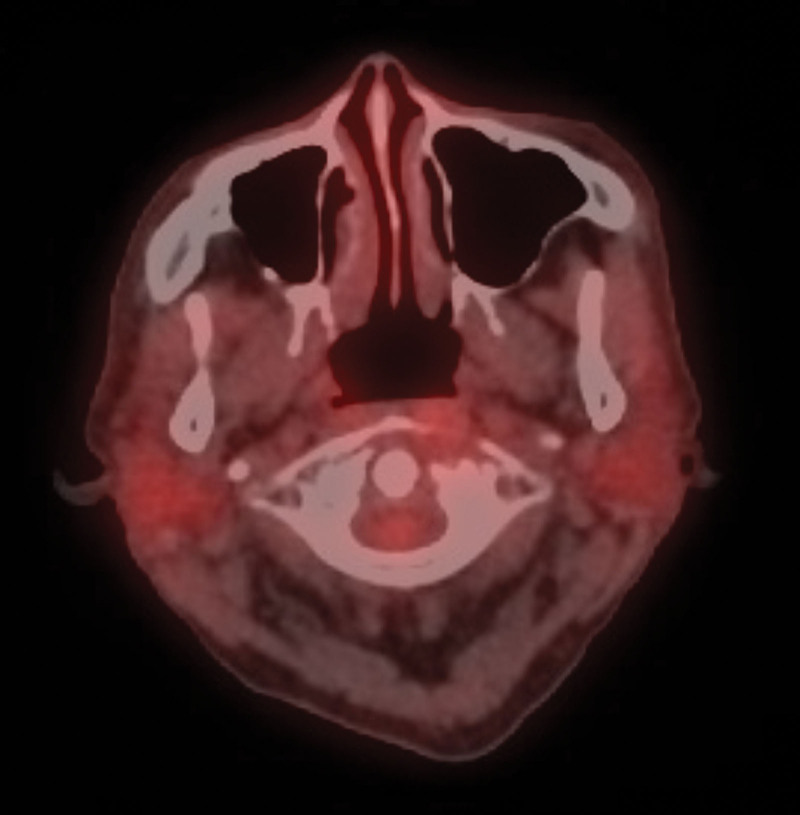
PET CT showed no evidence of FDG-avid lesion. CT = computed tomography, FDG = fluorodeoxyglucose, PET = positron emission tomography.

A 52-year-old Asian male presented with an 8-month history of a sore throat. Nasopharyngoscopy revealed a right-sided hypopharyngeal tumor. Biopsy revealed squamous cell carcinoma. The patient was diagnosed with hypopharyngeal cancer, which was observed over the right hypopharynx on computed tomography (CT) scan (Fig. 1). The clinical stage was T2N0M0, stage II. The patient underwent concurrent chemoradiotherapy (CCRT) with a radiation dose of 69.96 Gy in 33 fractions (Fig. 2). The chemotherapy schedule was concurrent weekly cisplatin at a dose of 40 mg/m^2^. After completing CCRT, biopsy of the previous tumor site showed no malignancy. After 2 months, the CT scan showed a complete response, and the positron emission tomography (PET) CT scan revealed no evidence of fluorodeoxyglucose (FDG)-avid malignant lesions (Fig. 3). Clinical complete response was confirmed based on these findings. The patient underwent regular follow-up and was disease-free for 2 years. However, 2 years later, a CT scan revealed a new solitary osteolytic lesion over the left anterior arch of the C1 vertebra. The patient did not have any neck pain or other symptoms. Magnetic resonance imaging (MRI) revealed a solitary enhancing lesion with normal marrow replacement and cortical bone breakthrough in the left anterior arch of the C1 vertebra (Fig. 4). PET CT revealed new uptake over the C1 vertebra. Solitary bony metastasis over the C1 vertebra was suspected. The patient refused biopsy of the C1 vertebral lesion because of the risk of the procedure. The patient opted for radiotherapy for the metastatic lesion in the C1 vertebra. However, because of the previous radiation dose delivery over the spinal cord and brainstem (40 and 37 Gy, respectively) during the initial disease, stereotactic fractionated radiotherapy (SFRT) with HA-VMAT has been suggested to limit the dose to critical organs, including the brainstem and spinal cord. The prescribed dose of radiotherapy was 25 Gy in 5 fractions. The patient experienced no acute adverse effects during or after RT. Four months later, PET CT showed no evidence of FDG-avid lesion (Fig. 5). MRI revealed regression of the metastatic tumor lesion over the C1 vertebra. The patient was disease-free and no late toxicity was observed after 9 months.

### 2.2. Radiotherapy

**Figure 6.**
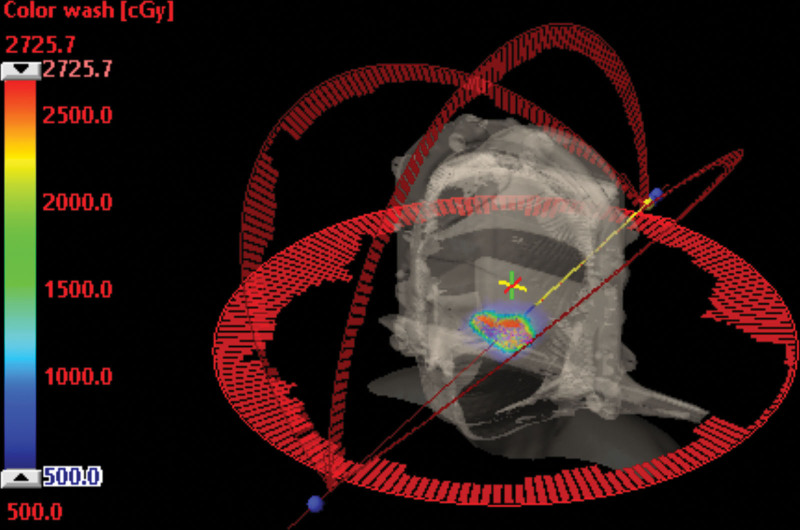
The HA-VMAT plan used 1 coplanar full arc and 3 noncoplanar partial arcs. VMAT = volumetric modulated arc therapy.

**Figure 7.**
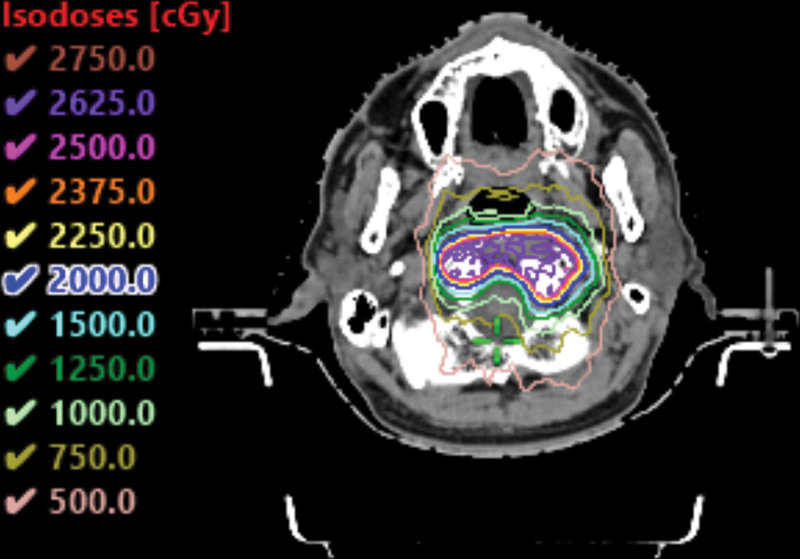
The isodose curve showed a rapid dose fall-off and highly conformal dose distribution.

**Figure 8.**
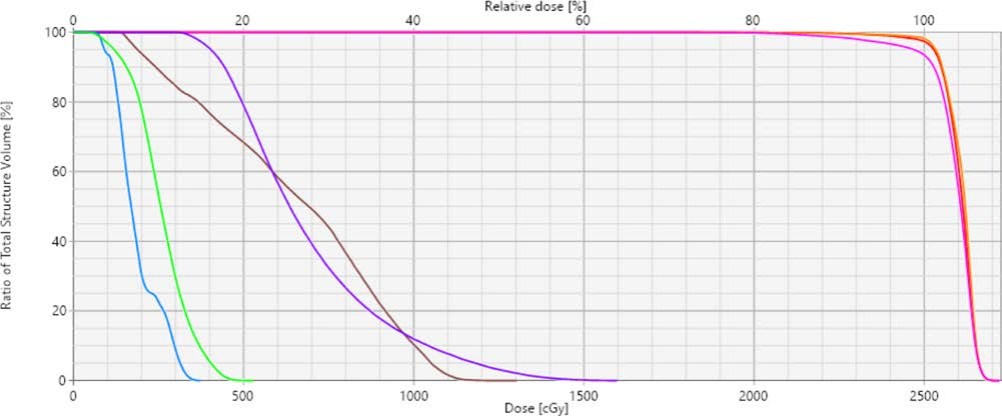
Dose-volume histograms for the target and organs at risk: GTV (orange line), CTV (red line), PTV (pink line), brainstem (purple line), spinal cord (brown line), mandible (green line), temporal lobe (blue line). CTV = clinical target volume, GTV = gross tumor volume, PTV = planning target volume.

For HA-VMAT planning, the patient was immobilized using the Qfix Encompass^TM^ SRS Immobilization System (Qfix, Avondale, PA). CT imaging (1-mm slice thickness) was performed. MRI scans were registered on the CT images for tumor delineation. The gross tumor volume (GTV) was defined using T1-weighted MRI. A margin of 5 mm was added to the GTV to generate the clinical target volume (CTV), and a margin of 2 mm was added to the CTV to generate the planning target volume (PTV). The PTV was 14 cm^3^. Automated noncoplanar HA-VMAT planning was performed for the patient. The goal was to provide a highly conformal dose to the target and minimize dose delivery to critical organs because of previous radiotherapy. A dose of 25 Gy in 5 fractions was prescribed to 98% of the PTV, which received 98% of the dose. The constraint of the spinal cord was 14 Gy and the constraint of the brainstem was 18 Gy. The HA-VMAT plan used 1 coplanar full arc and 3 noncoplanar partial arcs (Fig. 6). The isodose curve showed a rapid dose fall-off and highly conformal dose distribution (Fig. 7). After optimization, the actual coverage of the PTV and GTV was 93.4% and 98.2%, respectively. Dose-volume histograms for the target and organs at risk were shown (Fig. 8). Quality assurance was performed prior to treatment. Verification with pretreatment cone-beam CT was performed for every fraction of the treatment. The treatment was successfully completed.

## 3. Discussion

To the best of our knowledge, there have been no reports on the clinical outcomes of patients with oligometastatic head and neck cancer treated with HA-VMAT. The patient in this case had received high-dose radiation during the initial CCRT (upper spinal cord: 40 Gy, brain stem: 37 Gy), and reirradiation for bone metastasis in the C1 vertebra is quite challenging because of the limited tolerance dose of the brainstem and upper spinal cord. A new solution, HA-VMAT, was used to treat this patient. The HA-VMAT plan had a highly conformal dose distribution and a rapid dose fall-off. Good tumor coverage was achieved, but with sparing of the critical organs. The tumor response was favorable after treatment. No acute or late toxicity was observed.

Patients with oligometastatic head and neck cancer have a better prognosis than those with widespread metastases. Aggressive treatment options such as stereotactic body radiation therapy have been correlated with better survival.^[5]^ Our patient had a solitary bone metastasis over the C1 vertebra and was expected to have a good prognosis after ablative radiotherapy. We attempted to deliver moderate-to-high radiation doses to the tumor without causing toxicity to nearby critical organs. In our previous experience with reirradiation for head and neck cancer using intensity-modulated radiation therapy (IMRT) or conventional VMAT, it was difficult to achieve good tumor coverage and sparing of critical organs. In these situations, newer techniques such as HA-VMAT are ideal.

VMAT has a shorter treatment time than IMRT. Panet-Raymond et al^[6]^ reported that noncoplanar VMAT and IMRT techniques had better sparing of contralateral retina than coplanar VMAT and IMRT techniques for frontotemporal high-grade glioma (equivalent uniform doses: 15.36 and 15.96 Gy vs 22.05 and 18.79 Gy, *P* = .029). HA-VMAT minimized the workload, including automated settings for collimator angles, location of the isocenter, and arrangement of noncoplanar fields. Ohira et al^[1]^ demonstrated that HA-VMAT plans had higher conformity and rapid dose fall-off than conventional VMAT plans (conformity index: 0.93 vs 0.90, *P* = .01; gradient index: 3.06 vs 3.91, *P* < .01), which was also observed in our case.

HA-VMAT was initially used for the treatment of brain metastases. Ohira et al^[1]^ reported that HA-VMAT could be a new treatment option for single and multiple brain metastases in patients undergoing SRS. Two studies have shown that SRS with HA-VMAT is safe and effective for the treatment of brain metastases.^[2,7]^ A recent study reported that HA-VMAT provided better brain sparing than CyberKnife® in the treatment of multiple cranial metastases. Total brain V18Gy and V12Gy were 11 and 20.2 cm^3^ for HA-VMAT vs 18 and 34.1 cm^3^ for CyberKnife (*P* < .001).^[8]^ Recently, HA-VMAT has been used to treat nonbrain lesions. Inui et al^[3]^ demonstrated that HA-VMAT provided significantly lower mean brain doses than conventional VMAT for the treatment of scalp angiosarcoma (12.63 ± 3.31 Gy vs 17.11 ± 5.25 Gy, *P* = .005). Woods et al^[9]^ reported that in the treatment of head and neck cancer, HA-VMAT enabled significant PTV dose escalation compared to conventional VMAT planning (56.3 Gy vs 40.6 Gy, *P* < .001). Another study on locally recurrent head and neck cancer showed that HA-VMAT provided a highly conformal dose distribution and excellent sparing of critical organs.^[10]^ However, patients with hypopharyngeal cancer were excluded from this study. A recent study on SFRT for cervical metastatic spinal tumors revealed that HA-VMAT had a significantly higher target coverage, measured by GTV, than conventional VMAT (D_99%_: 89.3% ± 8.9% vs 84.2% ± 9.6%, *P* = .004).^[11]^ However, this study did not report the primary tumor site and did not include the C1 vertebra. One study on conventional radiotherapy for maxillary sinus cancer showed that HA-VMAT plans provided better target coverage than VMAT plans (D_99%_: 62.7 ± 2.1 Gy vs 61.9 ± 2.4 Gy, *P* = .009).^[4]^ Our case is the first to report the clinical outcomes of SFRT with HA-VMAT for hypopharyngeal cancer with C1 vertebral metastasis.

According to Soltys et al,^[12]^ the estimated prescription dose of stereotactic body radiation therapy for spinal metastases required to achieve a 2-year local control rate of 80% was 33 Gy in 5 fractions. In our case, the prescription dose was 25 Gy in 5 fractions because of concerns about toxicity to the spinal cord and brainstem. The previous course of radiotherapy delivered 40 and 37 Gy to the upper spinal cord and brainstem, respectively. An international consensus has recommended cumulative dose of 58.5 and 70.2 Gy across 2 doses of radiotherapy for the spinal cord and brainstem, respectively.^[13]^ In our case, after calculating the biologically effective dose, the constraints of the spinal cord and brainstem were limited to 14 and 18 Gy, respectively, during the second course of radiotherapy. After optimization, the actual coverage of the PTV and GTV was 93.4% and 98.2%, respectively, which was acceptable considering the tolerance doses of the spinal cord and brainstem. The patient had no acute or late toxicity. Dysphagia, odynophagia, and neck pain were not observed. Tumor regression was performed using imaging. Because the follow-up period was only 9 months in this study, further long-term follow-up to observe disease control and late toxicity is essential.

## 4. Conclusion

To the best of our knowledge, this is the first case report on the clinical outcomes of HA-VMAT for previously irradiated hypopharyngeal cancer with solitary recurrence over the C1 vertebra. HA-VMAT achieved a highly conformal dose distribution with excellent sparing of the critical organs. There was a favorable initial clinical response with no toxicity. Long-term follow-up is essential in such cases.

## Author contributions

**Conceptualization:** Chia-Hui Lin.

**Writing—original draft:** Chia-Hui Lin.

**Writing—review & editing:** Chia-Hui Lin.

**Data curation:** Jenny Que.

**Investigation:** Jenny Que.

**Supervision:** Sheng-Yow Ho.

**Validation:** Sheng-Yow Ho.
